# Hepatoprotective effects of brown algae *Sargassum boveanum* on bile duct-ligated cholestasis in rats are mediated by modulating NF-κB/TNF-α and Nrf2/HO-1 gene expression

**DOI:** 10.22038/AJP.2023.21970

**Published:** 2023

**Authors:** Zeinab Janahmadi, Hajar Jaberie, Abdolhamid Esmaili, Iraj Nabipour

**Affiliations:** 1 *Persian Gulf Marine Biotechnology Research Center, The Persian Gulf Biomedical Sciences Research Institute, Bushehr University of Medical Sciences, Bushehr, Iran*; 2 *Department of Biochemistry, School of Medicine, Bushehr University of Medical Sciences, Bushehr, Iran*; 3 *Department of Pathology, Bushehr University of Medical Sciences, Bushehr, Iran*

**Keywords:** Sargassum, Bile duct ligation, Cholestasis, Rat, Oxidative stress, Inflammation

## Abstract

**Objective:**

The current study assessed hepatoprotective effects of *Sargassum boveanum* (*S. boveanum*) in cholestatic rats. To induce cholestasis, bile duct ligation (BDL) was utilized.

**Materials and Methods:**

Five groups of Sprague-Dawley rats including Sham and four BDL groups were assigned to receive vehicle (BDL-V) or ethanolic extract of *S. boveanum* at 100 (BDL-SE 100), 200 (BDL-SE 200) and 500 (BDL-SE 500) mg/kg/day for seven days.

**Results:**

BDL group receiving the vehicle (BDL-V) had substantially increased blood levels of alkaline phosphatase, aspartate aminotransferase, alanine aminotransferase, total, and indirect bilirubin in comparison to the sham group. *S. boveanum *significantly decreased these variables compared to the BDL-V group. Hepatic malondialdehyde and tumor necrosis factor-α (TNF-α) level, and nuclear factor kappa light chain enhancer of activated B cells (NF-κB) and TNF-α gene expression were higher in BDL-V rats compared to the sham group but these were reduced markedly in BDL groups receiving *S. boveanum* in comparison to the BDL-V group. BDL-V group had a significantly lower hepatic glutathione value, glutathione peroxidase (GPx) and superoxide dismutase (SOD) activity and gene expression of SOD, GPx, Nrf2, HO-1 in comparison to the sham group. *S. boveanum* prevented the decrease of these variables. The histopathological assay showed marked bile ducts proliferation, portal inflammation, and hepatocellular damage in the BDL-V group and *S. boveanum* administration remarkably reduced hepatic injury. Gas chromatography-mass spectroscopy (GC-MS) analysis revealed that *S. boveanum *ethanolic extract contained 39 active compounds.

**Conclusion:**

*S. boveanum* treatment significantly ameliorated cholestatic hepatic injury via anti-oxidative and anti-inflammatory effects.

## Introduction

Hepatic disease is an important medical problem and main cause of global mortality and morbidity (Tsochatzis et al., 2014 ▶). Cholestasis is a clinical syndrome of liver injury resulting from bile flow impairment or defect in bile formation which is associated with pile of bile acids in systemic circulation and the liver (Li and Apte, 2015 ▶).

Bile duct ligation (BDL) is an established animal model that induces obstructive cholestasis. The BDL experimental model mimics the stereotypical histopathological phenotypes as in human cholestasis (Mariotti et al., 2018 ▶; Van Campenhout et al., 2019 ▶). The ligation of bile ducts was shown to be associated with increased liver damage serum markers like alkaline phosphatase (ALP), aspartate transaminase (AST), alanine transaminase (ALT), and bilirubin (Cabrera-Rubio et al., 2019 ▶). Even though the mechanism of cholestatic liver damage is not fully understood, inflammation and oxidative stress are known to play an influential role in liver damage. The bile acid accumulation induces inflammatory reactions and oxidative stress, leading to the progression of bile duct and hepatocellular injury, cirrhosis, and hepatic fibrosis (Copple et al., 2010 ▶; Kosters and Karpen, 2010 ▶). 

Oxidative stress is a key factor in cholestatic hepatic damage pathogenesis. According to human studies and experimental models of cholestasis, oxidative stress substantially contributes to the cholestasis-induced liver damage (Copple et al., 2010 ▶). Cholestasis is related to decreased activity of antioxidant systems specified by reduced hepatic glutathione (GSH), glutathione peroxidase (GPx), superoxide dismutase (SOD), and catalase (CAT), and increased formation of hepatic malondialdehyde (MDA) (Pastor et al., 1997 ▶; Grattagliano et al., 2014 ▶). 

Nuclear factor erythroid 2-related factor 2 (Nrf2) protein has a vital function in overall antioxidant response regulation. Previous studies indicated that Nrf2 activation can alleviate liver damage. Nrf-2 upregulates anti oxidative genes expression thus protects the liver from hepatotoxicity (Weerachayaphorn et al., 2012 ▶; Wang et al., 2014 ▶; Han et al., 2018 ▶; Zong et al., 2019 ▶). 

The pathogenesis of cholestatic liver disease is thought to be largely dependent on inflammatory reactions and oxidative stress via Nrf2 signaling pathway suppression stimulates the NF-κB which results in inflammation (Lin et al., 2019 ▶). NF-κB is known as a pivotal regulator of inflammatory response. NF-κB signaling pathway is reported to be the main factor in inducing the secretion of proinflammatory cytokines such as tumor necrosis factor-α (TNF-α) during the hepatic disease progression (Liu et al., 2001 ▶). 

Nearly over 70 % of earth is covered by water and marine organisms are a huge resource of biologically active compounds. Among marine organisms, marine algae comprise a wide range of active natural compounds with numerous biological effects. *Sargassum*, a brown alga belonging to the Sargasseae family, is prevalent in tropical and subtropical areas. *Sargassum* species are an abundant source of various compounds such as terpenoids, polysaccharides, polyphenols, sargachromenol, sargaquinoic acids, plastoquinones, steroids, glycerides, and carotenoids (Yende et al., 2014 ▶). Therefore, *Sargassum*, as a medicinal food, exerts numerous pharmacological effects such as antioxidant, neuroprotective, anticancer, anti-inflammatory, antidiabetic, and hepatoprotective effects (Yende et al., 2014 ▶; Palanisamy et al., 2018 ▶). 

This study aimed to investigate *S.* *boveanum* effects in a rat model of cholestasis. It also explored the *Sargassum*’s effects mediated by antioxidative and anti-inflammatory actions, and its role in the modification of NF-κB/TNF-α and Nrf2/HO-1 molecular pathways. 

## Materials and Methods

**Preparation of** ***S. boveanum*** **ethanolic** **Extract**

*S. boveanum *was collected from the Persian Gulf shores of Bushehr province, Iran and identified by a plant taxonomist and voucher specimen coded as 2663. The seaweed was cleaned using distilled water and air-dried in the shade at room temperature. The material was grounded to powder and then macerated with 95% ethanol over three days with occasional shaking. This procedure was repeated three times and after that, the extract was concentrated by rotary evaporator at reduced pressure at 40°C. The extract was kept at 4°C until further analysis.

**Measurement of total antioxidant activity, total phenolic and flavonoid content**

The total antioxidant activity of *S. boveanum* ethanolic extract was analyzed by cupric reducing antioxidant power (CUPRAC) assay (Apak et al., 2008 ▶). The antioxidant activity was measured in mg ascorbic acid equivalent/g of plant extract (mg AA/g). The Folin-Ciocalteu method, which has been developed by McDonald et al. (McDonald et al., 2001 ▶), was used to examine the total phenolic content. The results are reported as mg gallic acid equivalent/g of plant extract using gallic acid as the standard. The aluminum chloride (AlCl_3_) colorimetry test was used to determine the total flavonoid content of the extract. The total flavonoid content is reported as mg quercetin equivalent/g of plant extract (mg QUE/g), with quercetin being used as a standard (Chang et al., 2002 ▶).

**Gas chromatography-mass spectroscopy (GC-MS) analysis**

The GC-MS analysis of *S. boveanum* ethanolic extract was carried out by 7890B Agilent GC-MS system coupled with HP-5MS UI column (30 m×0.25 mm ID, 0.5 μm film thickness). EI mass spectra (m/z 50-500) were acquired using electrons with energies of 70 eV and 0.5 mA of filament emission. Helium gas (99.9999% purity) was used as the carrier gas (flow: 0.8 ml/min). After injecting the sample and waiting for three minutes, the GC oven temperature was scheduled to rise by 5°C per minute and hold at 250°C for ten minutes. The gas chromatograph injection port, ion source, and transfer line of 5977MSD were maintained at 240, 220, and 250°C respectively. A spectral library of the 2015 edition of NIST (National Institute of Standards and Technology) used for identifying the compounds.

**Animals**

Thirty-two male Sprague-Dawley rats weighing 200-250 g were provided by the Laboratory Animal Breeding Center, Bushehr University of Medical Sciences (Bushehr, Iran). Animals were maintained under conventional conditions at a temperature of 20-24°C, 25-35 % humidity with unrestricted access to water and food. All the procedures were done in accordance with the principles enacted by the ethics Committee for Care and Use of Animals of Bushehr University of Medical Sciences based on approval certificate number (IR.BPUMS.REC.1396.132).

**Surgical procedure**

Ketamine (60 mg/kg) (Rotexmedica, Germany) and xylazine (10 mg/kg) (Alfasan, Holland) were administered intraperitoneally to anesthetize the animals. The bile duct was isolated after a midline incision. It was then ligated by double ligatures by means of silk suture. Finally, the bile duct was cut between two ligatures. The sham operation included midline laparotomy as well as the identification and manipulation of the bile duct without ligation. 

**Experimental design**

After the operation, the rats were placed into five groups containing 6-7 rats in each: a sham-operated group treated with 1 ml of distilled water/day as vehicle (Sham-V), a group with the bile duct ligation treated with vehicle (BDL-V), and three groups with the bile duct ligation treated with *S. boveanum* ethanolic extract at 100 (BDL-SE 100), 200 (BDL-SE 200), and 500 (BDL-SE 500) mg/kg/day for seven days. Ethanolic extract *S. boveanum* was dissolved in distilled water. The vehicle or *S. boveanum* extract was administered by oral gavage for seven days. 

**Sampling**

On day eight, the animals were anesthetized with ketamine and xylazine; their blood samples were taken from abdominal aorta. Then, their livers were dissected out and split into three pieces after being washed with cold normal saline. The first and second portion were kept frozen at -80°C for biochemical analysis of oxidative stress and quantifying genes expression. The third portion was placed in 10% formalin solution to be fixated and prepared for histopathological purposes. The obtained blood samples were permitted to clot for 30 min at room temperature. The samples were then centrifuged at 3000 g for 15 min to separate the serum. They were kept frozen at -80°C until analysis. 

**Tissue homogenate preparation**

A portion of the liver tissue was homogenized (10% w/v) in ice-cold phosphate buffered saline using a homogenizer (IKA Werke Ultra-Turrax T25 basic homogenizer, Germany) to obtain homogenate. The homogenate was centrifuged at 4000 g at 4°C for 20 min, then, the resultant supernatant was collected and utilized for biochemical analysis. 

**Determination of liver function biomarkers**

Serum level of ALT, ALP, AST, and total and indirect bilirubin were analyzed using a Hitachi 902 chemistry autoanalyzer commercially kit purchased from Pars Azmun Co. (Iran). 

**Determination of oxidative stress biomarkers and antioxidant enzymes activity**

GSH concentration, MDA as oxidative stress indicators, and antioxidant enzymes including GPx and SOD were measured in hepatic tissue homogenates. All the assays were done using ZellBio GmbH (Germany) commercial kits following the manufacturer's instructions.

**Proinflammatory cytokine TNF-α level analysis**

TNF-α concentration in hepatic tissue homogenates was examined using a commercial ELISA kit purchased from Diaclone (France) following the instructions of the manufacturer. 

**Quantitative real time PCR: RNA isolation and RT-qPCR**

Following the protocol of the manufacturer, RNA was extracted from the liver tissue using Trizol (Invitrogen, Carlsbad, CA, USA) and the total concentration of RNA was measured at 260 nm wavelength with Nano drop 1000 spectrophotometer (Thermo Fisher Scientific, Inc.). Next, cDNA synthesis was carried out with 5 μg of total RNA according to the instructions of the manufacturer (Thermo Fisher Scientific, Waltham) in a 20-μl final volume. qPCR was also carried out using a Corbet Rotor-Gene 6000 rotary analyzer (Corbett, Australia) with a 15-µl PCR mixture that involved 1 µl cDNA, 7.5 µl 2X SYBR^®^ Green PCR low rox Master mix (Amplicon, Denmark), 1 µl forward primer of 5 pmol, 1 µl reverse primer of 5 pmol and 4.5 µl nuclease-free water. The reactions were initiated in a heat denaturation step at 95°C for 10 min, followed by 40 cycles at 95°C for 10 sec, at 60°C for 30 sec, and 72°C for 30 sec. The synthesis of the primer pairs sequences (Table 1) was done by Metabion (Germany). The levels of *GAPDH* were employed for normalization gene expression and fold change of each transcript was determined by the ΔΔC*t *method (Livak and Schmittgen, 2001 ▶). 

**Histopathological examination**

 The liver tissues were first immersed in 10% formalin and then, paraffin was used to encase them. Hematoxyline and eosin (H&E) was used to stain the sections, and then, the sections were examined under light microscope for hisopathological evaluation. The histopathological analysis was performed qualitatively for following parameters: integrity of lobular architecture, portal inflammation, and proliferation portal ducts (Kim, Lee et al. 2012 ▶, Ali, Azouz et al. 2018 ▶). 

**Statistical analysis**

The data are presented as mean±SEM. To examine the statistical significance, the experimental data were evaluated using One-Way Analysis of Variance (ANOVA), which was followed by Duncan's Multiple Range test for pairwise comparisons. A level of p value <0.05 was considered acceptable for statistical significance. The SigmaPlot statistical and graphical software version 11.0 (San Jose, CA, USA) were used to analyze the data.

## Results

**Antioxidant activity, and flavonoid and total phenolic contents**

The antioxidant activity of *S. boveanum* ethanolic extract was 1344.7±90.3 mg AA/g. The flavonoid and total phenolic contents of the extract were 1986.4±151.3 mg QUE/g and 107.2±5.8 mg GA/g, respectively.

**GC-MS composition analysis**

**Figure 1:**
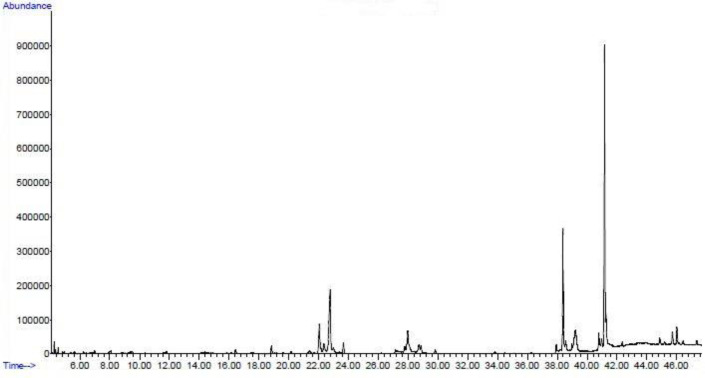
GC-MS chromatogram of *S. boveanum* ethanolic extract

GC-MS chromatogram of ethanolic extract of *S. boveanum* is presented in Figure 1. A total of 39 compounds were found. The compounds name with their retention time (RT), chemical structure, and proportion (%) in the ethanolic extract of *S. boveanum* are presented in Table 2. Octadecanoic acid 2, hydroxyl-1-(hydroxymethyl) ethyl ester showed the highest peak followed by n-hexadecanoic acid, hexadecanoic acid 2,3-dihydroxy propyl ester, di-n-octyl phthalate, and 9,12-octadecadienoic acid (Z, Z) in this chromatogram indicating their higher proportion compared with other compounds. 

**Table 1:** Sequence of primers for quantitative real time PCR

**Gene**	**Forward primer**	**Reverse primer**	**Size (bp)**
***Nrf-2***	5' CACATCCAGACAGACACCAGT 3*'*	5' CTACAAATGGGAATGTCTCTGC 3'	121
***HO-1***	5' ACAGGGTGACAGAAGAGGCTAA 3'	5' CTGTGAGGGACTCTGGTCTTTG 3'	107
***SOD***	5' ACACAAGGCTGTACCACTGC 3'	5' CCACATTGCCCAGGTCTCC 3'	103
***GPx***	5' GTCCACCGTGTATGCCTTCTCC 3'	5' TCTCCTGATGTCCGAACTGATTGC 3'	218
***NF-κB***	5' GGCAGCACTCCTTATCAA 3'	5' GGTGTCGTCCCATCGTAG 3'	249
***TNF-α***	5' CCCACGTCGTAGCAAACCACCA 3'	5' CCATTGGCCAGGAGGGCGTTG 3'	79
***GAPDH***	5'TACCCACGGCAAGTTCAACG 3'	5'CACCAGCATCACCCCATTTG 3'	122

**Table 2:** Components identified from the *S. boveanum* ethanolic extract of in GC-MS analysis

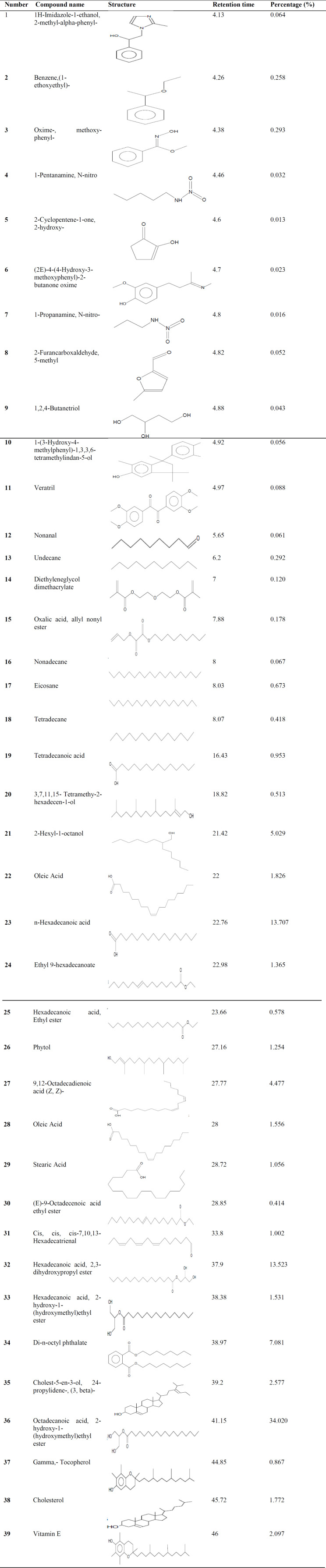

**Serum biochemical factors assessment**

As revealed in Table 3, BDL significantly augmented serum ALT (386.6±23.8 vs 65.0±6.0 U/L), AST (1247±137.1 vs 122.6±12.9 U/L), ALP (1269±78.9 vs 316.5±34.5 U/L), total bilirubin (9.373±0.605 vs 0.158±0.005 mg/dl) and indirect bilirubin (2.179±0.209 vs 0.11±0.009 mg/dl) levels in comparison to sham-V group (p<0.001). *S. boveanum* ethanolic extract (100, 200 and 500 mg/kg) significantly reduced serum ALT (−38.05%, −44.24% and −48.67% respectively, p<0.001), AST (−29.4%, −45.22% and −56.93% respectively, p<0.05) and indirect bilirubin (−19.59%, −26.93% and −21.61% respectively, p<0.05) levels compared to the BDL-V rats. Administration of* S. boveanum* ethanolic extract (200 and 500 mg/kg) significantly reduced serum ALP (−26.16% and −28.95% respectively, p<0.001) and total bilirubin (−12% and −34.29% respectively, p<0.05).

**Oxidative stress biomarkers**

**Figure 2:**
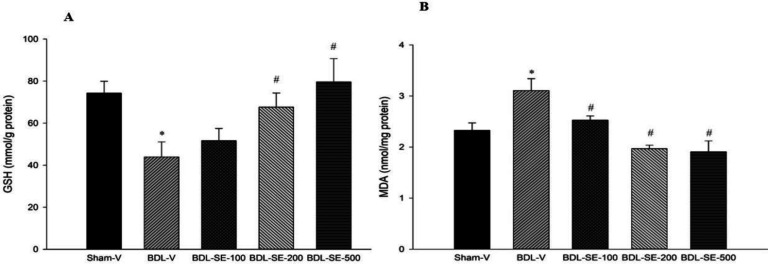
Oxidative stress biomarkers in liver tissue. GSH glutathione; MDA malondialdehyde. Sham-V Sham group receiving 1 ml distilled water/day as vehicle; BDL-V bile duct ligation group receiving 1 ml distilled water/day as vehicle; BDL-SE 100, BDL-SE 200, and BDL-SE 500 bile duct ligation groups receiving *S. boveanum* ethanolic extract at 100 mg/kg/day, 200 mg/kg/day, and 500 mg/kg/day respectively. Data are expressed as means±SEM (n=6-7). *Significant (p<0.05) difference from the Sham-V group; #Significant (p<0.05) difference form the BDL-V group

Hepatic content of MDA (1.33-fold) was increased significantly in the BDL-V group (p=0.004), along with a significant decrease in GSH level (−40.91%) in comparison to the sham group (p=0.021). *S. boveanum* ethanolic extract (100, 200 and 500 mg/kg) significantly attenuated MDA levels in comparison to the BDL-V group (−16.45%, −36.77% and −38.7% respectively, p<0.05). Administration of extract (200 and 500 mg/kg) after BDL significantly augmented GSH level (1.53 and 1.81 –fold increase, respectively, p<0.05, Figure 2).

**Table 3:** Serum levels of liver function biomarkers in all experimental groups

**Parameters**	**Sham-V**	**BDL-V**	**BDL-SE 100**	**BDL-SE 200**	**BDL-SE 500**
**ALT (U/ L)**	65.0±6.0	386.6±23.8^*^	239.5±33.9^##^	215.6±12.3^##^	202.3±41.7^##^
**AST (U/ L)**	122.6±12.9	1247±137.1^*^	880.5±83.8^#^	683.0±100.8^#^	539.6±93.3^#^
**ALP (U/L)**	316.5±34.5	1269±78.9^*^	1131.5±40.3	937.3±66.7^##^	901.6±36.7^##^
**Total Bilirubin (mg/dl)**	0.158±0.005	9.373±0.605^*^	8.237±0.344	7.405±0.443^#^	6.157±1.018^#^
**Indirect bilirubin (mg/dl)**	0.110±0.009	2.179±0.209^*^	1.752±0.097^#^	1.592±0.130^#^	1.708±0.091^ #^

Data are shown as Mean±SEM, N=6-7 each group. ALT alanine transaminase; AST aspartate transaminase; ALP alkaline phosphatase; Sham-V Sham group receiving 1 ml distilled water/day as vehicle; BDL-V bile duct ligation group receiving 1 ml distilled water/day as vehicle; BDL-SE 100 bile duct ligation group receiving S. boveanum ethanolic extract at 100 mg/kg/day; BDL-SE 200 bile duct ligation group receiving S. boveanum ethanolic extract at 200 mg/kg/day; BDL-SE 500 bile duct ligation group receiving S. boveanum ethanolic extract at 500 mg/kg/day.*Significant (p<0.001) difference form Sham-V group; #Significant (p<0.05) difference form BDL-V group; ## Significant (p<0.001) difference form BDL-V group

**Antioxidant enzymes activity and gene expression**

**Figure 3:**
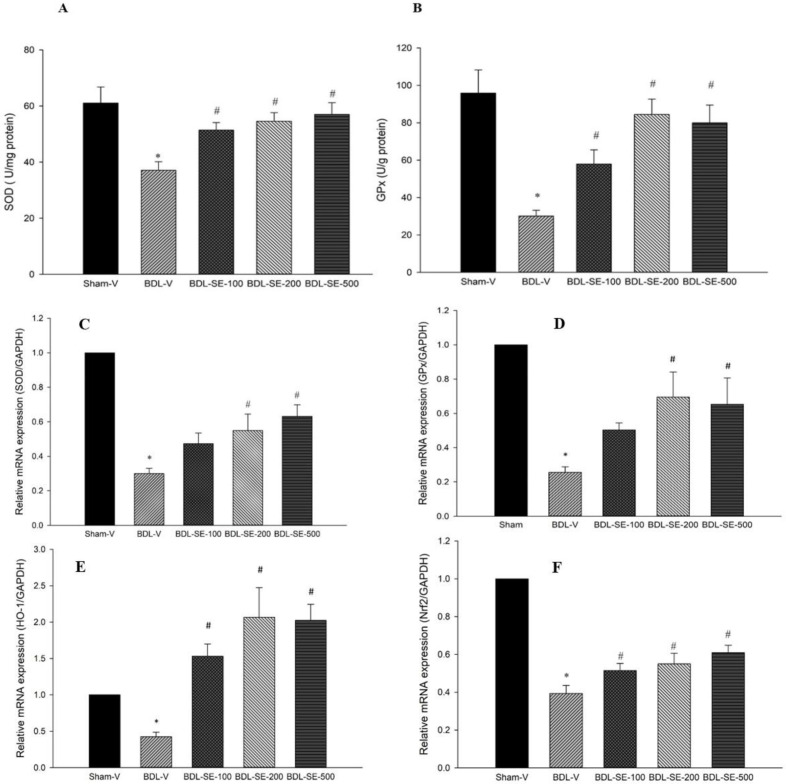
Antioxidant activity and gene expression. Hepatic level of SOD and GPx and mRNA expressions of *SOD*, *GPx*, *HO-1*, *Nrf-2* in the sham group (Sham-V), bile duct ligated group treated with vehicle (BDL-V), and bile duct ligated groups treated with *S. boveanum* ethanolic extract at 100, 200 and 500 mg/kg/day. Data are expressed as means ± SEM (n=6-7). *Significant (p<0.001) difference from the Sham-V group; #Significant (p<0.05) difference form the BDL-V group

As presented in Figure 3, ligation of bile duct significantly attenuated hepatic mRNA expression of *Nrf2* (−61%, p<0.001), *HO-1* (−58%, p<0.001**), ***SOD* (−70%, p<0.001) and *GPx* (−74%, p<0.001) along with significant decrease in activity of SOD (−39.27%, p<0.001) and GPx (−68.58%, p<0.001) in comparison to the sham-V group. Administration of *S. boveanum* ethanolic extract (100, 200 and 500 mg/kg) after ligation of bile duct markedly improved genes expression of *Nrf2* (1.3, 1.41 and 1.56-fold increase p<0.05, respectively), *HO-1* (3.64, 4.9 and 4.8 -fold increase p<0.05, respectively) and *SOD* (1.56, 1.83 and 2.1-fold increase p<0.05, respectively) and hepatic activity of SOD (1.38, 1.47 and 1.53 -fold increase p<0.05, respectively) and GPx (1.92, 2.8 and 2.65-fold increase p<0.05, respectively). Ethanolic extract of *S. boveanum* at doses of 200 and 500 mg/kg also caused significant increases in *GPx* gene expression in comparison to the BDL-V group (2.65 and 2.5-fold increase p<0.05, respectively).

**Hepatic content of TNF-α and gene expression of TNF-α and NF-κB**

**Figure 4:**
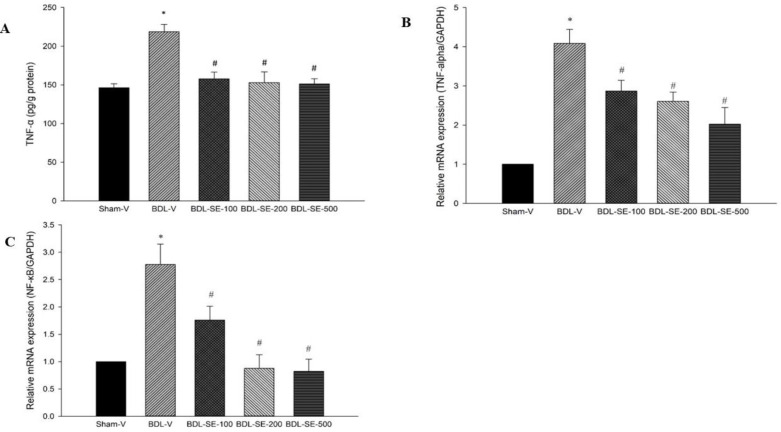
Hepatic level of TNF-α and mRNA expressions of *NF-κB*, and *TNF-α* in sham group (Sham-V), bile duct ligated group treated with vehicle (BDL-V), and bile duct ligated groups treated with *S. boveanum* ethanolic extract at 100, 200 and 500 mg/kg/day. Data are expressed as means±SEM (n=6-7). *Significant (p<0.001) difference from the Sham-V group; # Significant (p<0.05) difference form the BDL-V group

Hepatic content of TNF-α was significantly increased in BDL-V group (218.8±9.3 vs 146.3±5.2 pg/g) compared to sham-V group (p<0.001). Gene expression of *NF-κB* and *TNF-α* were elevated in the BDL-V group (2.77 and 4.08-fold increase respectively) in comparison to the sham-V group (p<0.001). Rats treated with ethanolic extract of *S. boveanum* (100, 200 and 500 mg/kg) after BDL significantly reduced hepatic level of TNF-α (−27.87%, −30.07% and 30.94% (p<0.05) , respectively) along with significant reduction in hepatic expression of *TNF-α* (−29.65, −36.27% and −50.49% p<0.05, respectively) and *NF-κB* (−36.46%, −68.59% and −70.39% p<0.05, respectively) in comparison to the BDL-V group (Figure 4).

**Histopathological studies**

**Figure 5:**
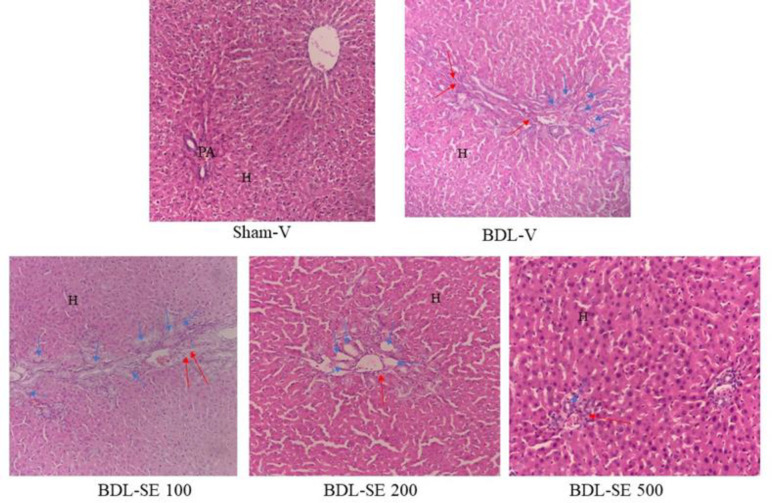
Histopathology of liver sections stained with hematoxylin and eosin for the Sham-V group receiving 1 ml distilled water/day as vehicle; BDL-V bile duct ligation group receiving 1 ml distilled water/day as vehicle; BDL-SE 100, BDL-SE 200, and BDL-SE 500 bile duct ligation groups receiving *S. boveanum* ethanolic extract at 100, 200, and 500 mg/kg/day, respectively. Rat livers showed marked proliferation of bile ducts and portal inflammation following BDL. Treatment with *S. boveanum* ethanolic extract ameliorated bile duct proliferation, portal inflammation, and preserved lobular architecture. All photos are presented at 100 × magnification. H: hepatocyte, PA: portal area, blue arrows indicate bile duct proliferation, red arrows indicate portal inflammation

The histopathological analysis of the liver in the Sham-V group showed a normal architecture without portal inflammation and proliferation of portal duct. In the BDL-V group the normal liver architecture was totally lost with severe portal inflammation and portal ductular proliferation. Treatment with *S. boveanum* ethanolic extract (200, 500 mg/kg/day) alleviated the BDL-induced liver damage and the histopathological changes including portal inflammation and proliferation of portal ducts were significantly ameliorated in the liver tissue (Table 4 and Figure 5). 

**Table 4:** Liver histopathological alterations (H&E) in all experimental groups

	**Lobular architecture**	**Portal inflammation**	**Portal ductular proliferation**
**Sham-V**	Preserved	-	-
**BDL-V**	Effaced	moderate	Severe
**BDL-SE 100**	Effaced	Mild to moderate	Moderate to severe
**BDL-SE 200**	Rather preserved	Mild	Moderate
**BDL-SE 500**	Preserved	Trace	-

Sham-V group receiving1 ml distilled water/day as vehicle; BDL-V bile duct ligation group receiving 1 ml distilled water/day as vehicle; BDL-SE 100, BDL-SE 200, and BDL-SE 500 bile duct ligation groups receiving *S. boveanum* ethanolic extract at 100, 200, and 500 mg/kg/day respectively.

## Discussion

The present findings illustrate that BDL results in cholestasis and* S. boveanum* ethanolic extract offers hepatoprotection in rats with cholestasis. Accordingly, the hepatoprotection offered by ethanolic extract of *S. boveanum* might be attributed to antioxidative and anti-inflammatory effects. 

In the current research, the ethanolic extract of *S. boveanum* was observed to strongly reduce Cu (II)-neocuproine to Cu(I)- neocuprine in CUPRAC method. This suggests that phenolic and flavonoid contents of the *S. boveanum* ethanolic extract can help scavenge free radicals (Moure et al., 2000 ▶; Jayaprakasha et al., 2008 ▶). It is widely known that flavonoids are among the main and effective antioxidant ingredients in plant foods (Velioglu et al., 1998 ▶). Thus, it is not surprising that *S. boveanum* with its potent phenolic antioxidant is effective in treating a variety of disorders connected to oxidative stress.

In the current research, the ligation of bile duct resulted in the deterioration of the liver integrity as indicated by increased serum levels of AST, ALT, ALP, and total and indirect bilirubin. These results are in accordance with human cholestasis (Hayat et al., 2005 ▶; Assimakopoulos et al., 2006 ▶) and experimental models of liver injury induced by BDL (Tag et al., 2015 ▶; Cabrera-Rubio et al., 2019 ▶) and CCl_4_ (Akbartabar Toori et al., 2015 ▶; Zarezade et al., 2018 ▶; Xu et al., 2020 ▶). Our findings demonstrated that treatment with ethanolic extract of *S. boveanum* improved all liver function biomarkers, a view supported by previous reports (Quintal-Novelo et al., 2018 ▶; Sohail et al., 2019 ▶). Corroborating with other studies (Abshagen et al., 2015 ▶, Sen et al., 2016 ▶), the histopathological findings of our study show that BDL was accompanied with bile duct proliferation, inflammation, and disruption of lobular architecture. We found that treatment with *S. boveanum* ethanolic extract could help to alleviate inflammatory cells infiltration and bile duct proliferation and preserve the liver architecture. These results are consistent with those of earlier research (Madkour et al., 2012 ▶; Mutawie and El-Naggar, 2013 ▶; Quintal-Novelo et al., 2018 ▶; Chale-Dzul et al., 2020 ▶). 

BDL could result in hepatic dysfunction through several mechanisms such as inflammatory cascades and oxidative stress. We also explored whether oxidative stress had any role in liver dysfunction. The present results show that BDL was linked to increased hepatic MDA content, decreased hepatic GSH content and SOD and GPx activity. It was also found that hepatic expression of SOD, GPx, HO-1 and Nrf2 was decreased in the BDL-V group; a finding demonstrated by previous studies (Orellana et al., 2000 ▶; Colares et al.; 2016 ▶). Our findings show that ethanolic extract of *S. boveanum* help increase hepatic GSH content and GPx and SOD activity and reduce hepatic MDA content. Our results also demonstrated that *S. boveanum* ethanolic extract enhanced hepatic expression of SOD, GPx, HO-1, and Nrf2 which is in harmony with previous studies. Several studies have examined the mechanism of hepatoprotective effects of *Sargassum* in various circumstances such as acetaminophen-induced hepatitis (Raghavendran et al., 2004 ▶; Hira et al., 2019 ▶), CCl_4_-induced liver damage (Altinok-Yipel et al., 2019 ▶; Chale-Dzulet al., 2020 ▶), and in HepG2 cell line (Lim et al., 2018 ▶), suggesting that the effects of *Sargassum* could be partially associated to the reduction of oxidative stress. Adjustment of cellular redox homeostasis is chiefly managed by the Nrf2/Keap1/ARE pathway. Cytoplasmic Nrf2 is normally joined to Kelch-like ECH associating protein 1 (Keap1) as its specific inhibitor. In oxidative stress situations, Nrf2 detaches from Keap1 and translocates into the nucleus. Subsequently, it combines with transcription factors. This complex binds to the ARE and stimulates the transcription of antioxidant genes, containing HO-1, Nqo1 (NADPH oxidoreductase 1) (Li et al., 2014 ▶). HO-1 plays as an effective cytoprotective via antioxidant and anti-inflammatory properties (Berne et al., 2012 ▶).

According to previously reported research, inflammatory processes can get involved in hepatic injury after BDL and BDL-induced oxidative stress activates inflammatory pathways such as NF- κB (Gäbele et al., 2009 ▶, Li et al., 2017 ▶). Hence, we measured the hepatic content of TNF-α and expression of TNF-α and NF-κB gene in the liver tissue. Our results indicated a noticeable increase in hepatic TNF-α content and NF-κB and TNF-α expression after BDL. Similar findings were observed in human cholestasis (Kosters and Karpen, 2010 ▶) and animal models of liver injury such as acetaminophen (Lee et al., 2019 ▶), CCl_4_ (Zhang et al., 2004 ▶; Wang et al., 2018 ▶; Wei et al., 2022 ▶), and BDL (Gabbia et al., 2018 ▶; Wei et al., 2019 ▶). Treatment with *S. boveanum* ethanolic extract significantly reduced TNF-α hepatic content and TNF-α and NF-κB gene expression. Similar findings have been reported in the literature, indicating anti-inflammatory effects of *Sargassum* in LPS-stimulated inflammation in RAW 264.7 cells (Yang et al., 2013 ▶; Kim et al., 2015 ▶; Jayawardena et al. 2019 ▶), Freund's complete adjuvant-induced arthritis model, carrageenan-induced peritonitis model (Neelakandan and Venkatesan, 2016 ▶), endothelin-1 stimulated human keratinocytes (Sah et al., 2013 ▶), and vascular inflammation (Gwon et al., 2017 ▶). Numerous studies have revealed that sargassum has anti-inflammatory properties that reduce the production of pro-inflammatory cytokines including TNF-α, interleukin-6, interleukin-1β while suppressing the NF-κB, cyclooxygenase-2, and inducible nitric oxide synthase pathways (Lee et al., 2013 ▶; Han et al., 2018 ▶; Kim et al., 2018 ▶; Jayawardena et al., 2019 ▶; Saraswati et al., 2019 ▶). Fatty acids such as stearic acid and its derivatives were discovered to be the main chemical components of ethanolic extract, according to the GC-MS study. Several studies reported that stearic acid shows anti-inflammatory effects. Previous studies indicated that saturated fatty acids have hepatoprotective effects in alcohol-induced hepatic damage (Nanji et al. 1997 ▶; Nanji et al., 2001 ▶) and stearic acid shows protective effects in cholestatic liver injury via anti-inflammatory effects and NF-κB suppression (Pan et al., 2010 ▶). 

To our knowledge, this is the first research to indicate that ethanolic extract of *S. boveanum* offers hepatoprotection in cholestatic rats by modifying Nrf2/HO-1 and NF-κB/TNF-α gene expression. Our study also had some limitations. One limitation of the current study was that the investigation was done on crude extract. Another limitation was the lack of protein expression analysis. It would be interesting to investigate the effects of major components of *S. boveanum *on protein expression of antioxidant and anti-inflammatory pathways.

In conclusion, ethanolic extract of *S. boveanum* is highly effective in preventing hepatic damage induced by BDL. The hepatoprotective effects of *S. boveanum* could be probably mediated by its ability to attenuate oxidative stress and suppression of inflammatory reactions induced by BDL as well as enhancement of Nrf2/HO-1 and downregulation of TNF-α/NF-κB signaling pathways.

## Conflicts of interest

The authors have declared that there is no conflict of interest.
